# Supplementary material for: The ϕPA3 phage nucleus is enclosed by a self-assembling 2D crystalline lattice
Source: Nat Commun. 2023 Feb 18;14:927. doi: 10.1038/s41467-023-36526-9 (PMC9938867; doi:10.1038/s41467-023-36526-9)
Supplement: Supplementary file 1 — Supplementary Information [file 41467_2023_36526_MOESM1_ESM.pdf]

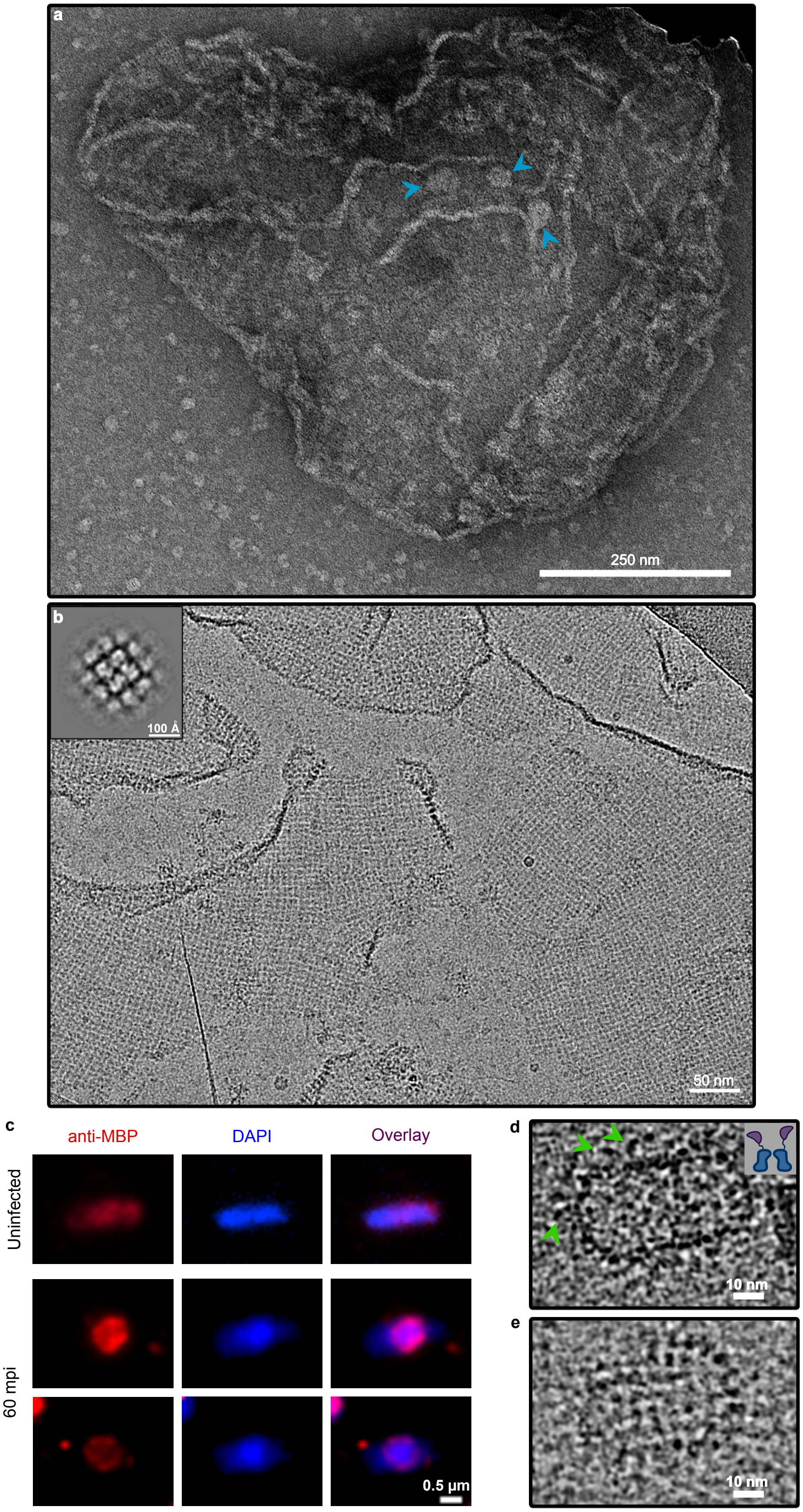


**Supplementary Fig. 1 | ϕPA3 PhuN assemblies. a,** Negative Stain micrograph of a phage nuclear shell fragment isolated from *P. aeruginosa* expressing 6XHisMBP-PhuN. The isolated species is about the size expected for an intact compartment. The lattice texture is visible as well as many wrinkles and folds. Smaller, round species are present on the surface (blue arrows), similar in appearance to Class II assemblies *in vitro*. (n = 2 for shell fragments, n = 1 for likely complete shell.) **b,** CryoEM micrograph of a shell fragment isolated from *P. aeruginosa* expressing 6XHisMBP-PhuN showing many shell fragments with a lattice texture, comparable to the lattices observed in main Figure 1. (n = 1). **c,** Immunofluorescence microscopy of *P. aeruginosa* cells expressing the 6XHisMBPGp53 fusion. The fusion protein integrates into the shell and localizes at the cell center around the phage DNA 60 minutes post infection. (n = 1). **d,** CryoEM images of *in vitro* PhuN assemblies. Densities consistent with the presence of MBP are visible on the exterior (green arrows). The inset shows a cartoon representation of a side-view of PhuN (blue) with MBP (purple). (n = 1). **e,** The assemblies show a lattice like surface. (n = 1).

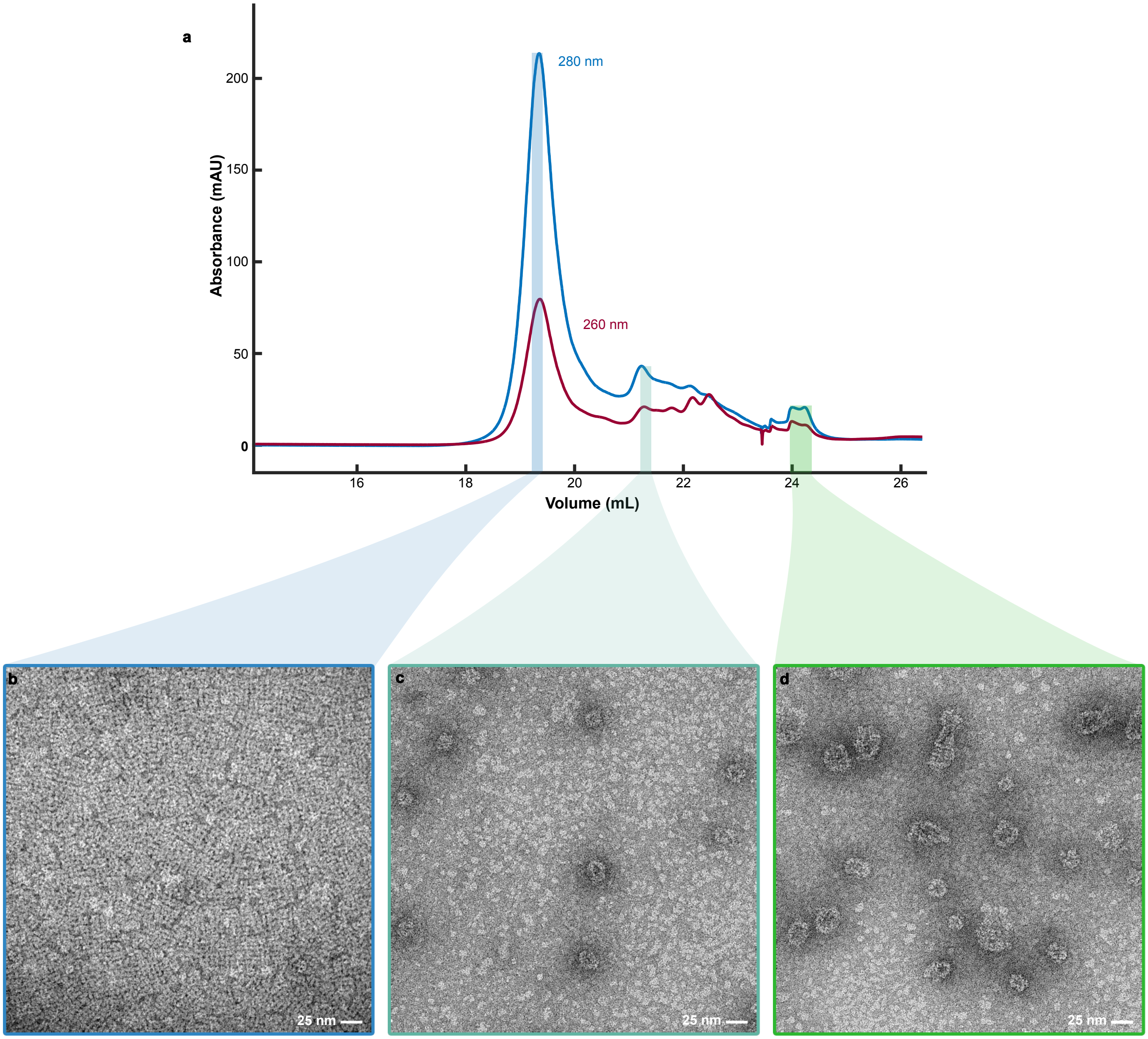


**Supplementary Fig. 2 | PhuN crystalizes at pH 6.5. a.** Anion exchange purification using a 1 mL GE MonoQ column at pH 6.5. Highlighted fractions correspond to micrographs b-d. **b,** Monomeric species in Class I form 2D polycrystalline assemblies when applied to negatively charged grids for negative stain EM. **c,** 25 nm mid-sized species present in Class II. **d,** Assemblies 25 nm and larger present in later elution in Class III. Samples prepared and imaged n = 3 times.

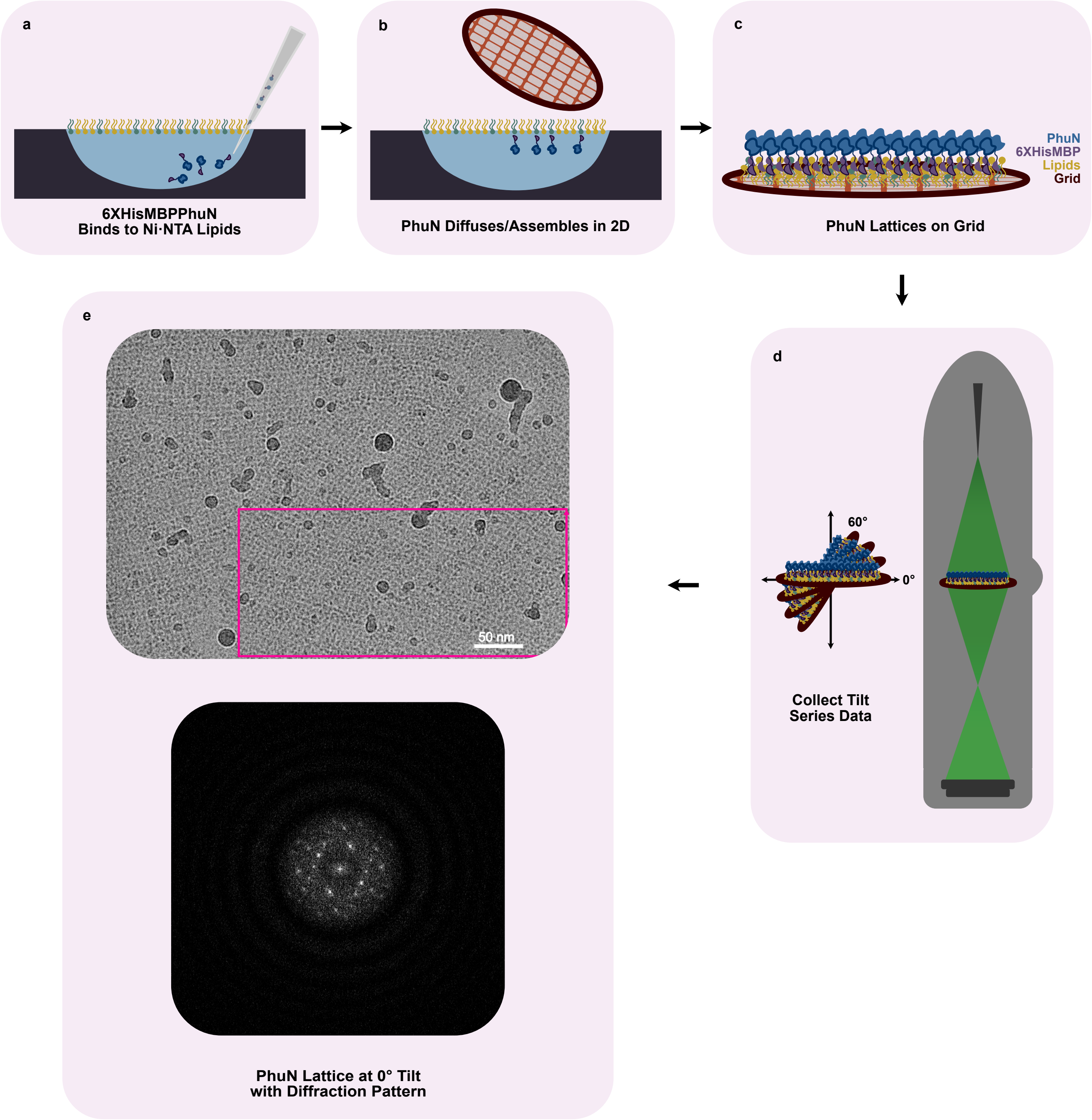


**Supplementary Fig. 3 | *in vitro* 2D crystal preparation. a,** 6XHisMBP-PhuN is injected into a buffer droplet with a pre-formed 21% Ni·NTA Lipid Monolayer. **b-c,** After incubation to allow for assembly, the PhuN arrays are adsorbed to a grid by touching the carbon side of the grid to the top of the lipid-containing droplet. These grids are then plunge frozen for CryoEM. **d,** Data is collected at fixed tilts ranging from 0-60°. **e,** 0° image from Fig. 1c shown with the diffraction patten corresponding to the region denoted with the magenta rectangle. (n = 1).

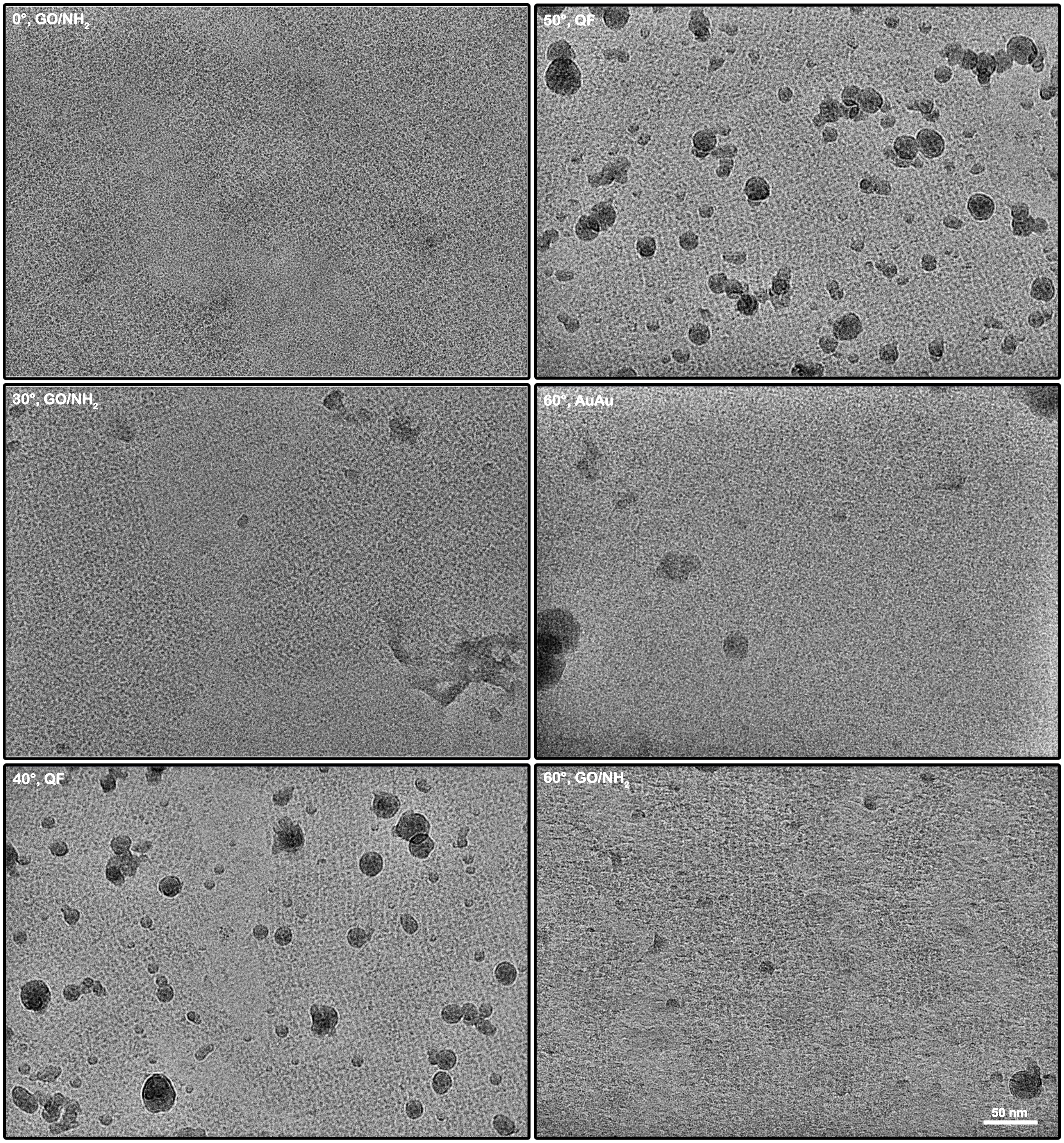


**Supplementary Fig. 4 | Example micrographs collected at the specified tilt angle.** Tilt angle is listed alongside the grid support used in each micrograph: Traditional Quantifoil (QF), Amino-functionalized graphene-oxide (GO/$NH_2$), UltrAuFoil (AuAu). The lattices often appear with bends and breaks as seen throughout these micrographs. Number of datasets collected: n(0°, GO/$NH_2$) = 3; n(30°, GO/$NH_2$) = 1; n(40°, QF) = 1; n(50°, QF) = 1; n(60°, AuAu) = 1; n(60°, GO/$NH_2$) = 3.

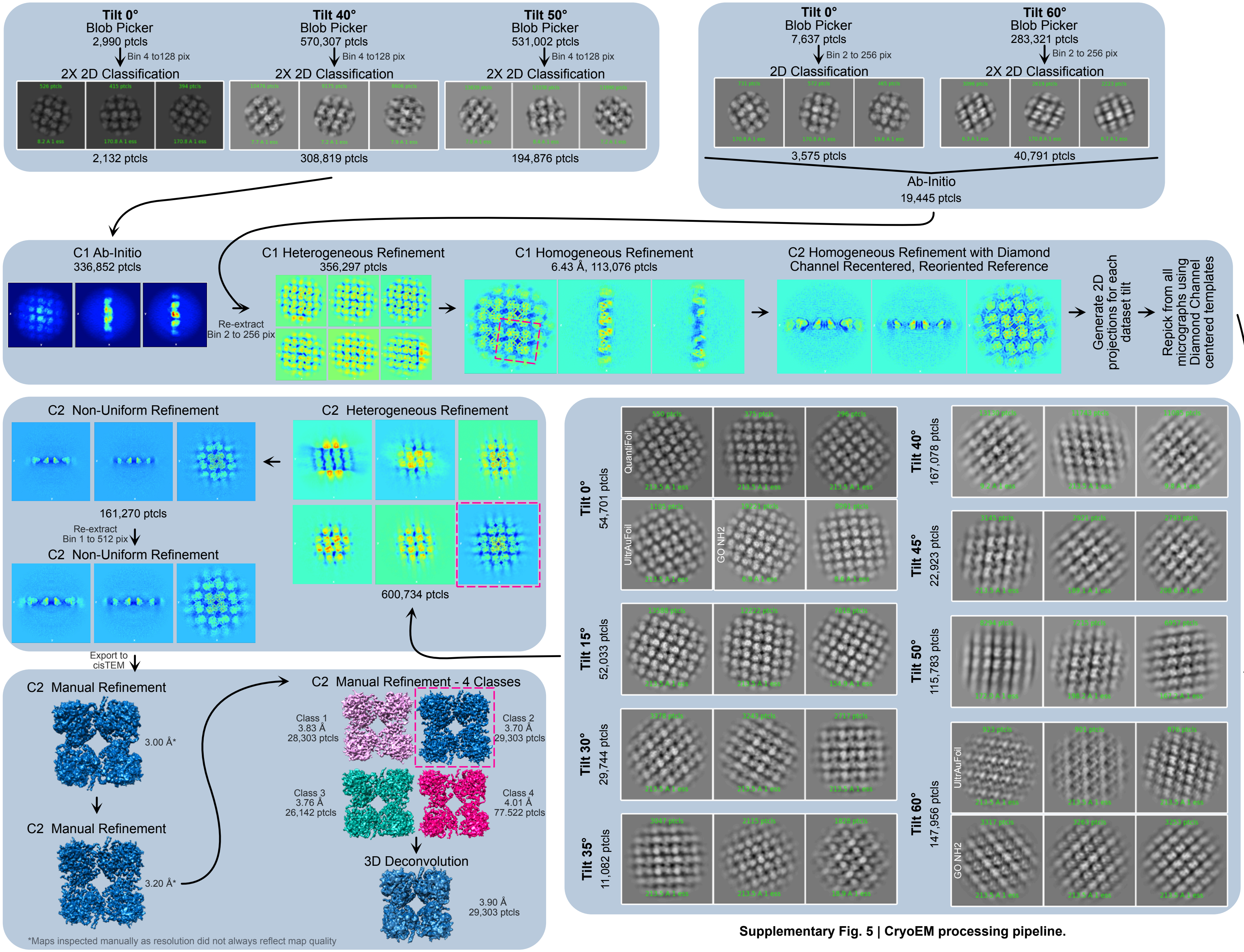


**Supplementary Fig. 5 | CryoEM processing pipeline.**

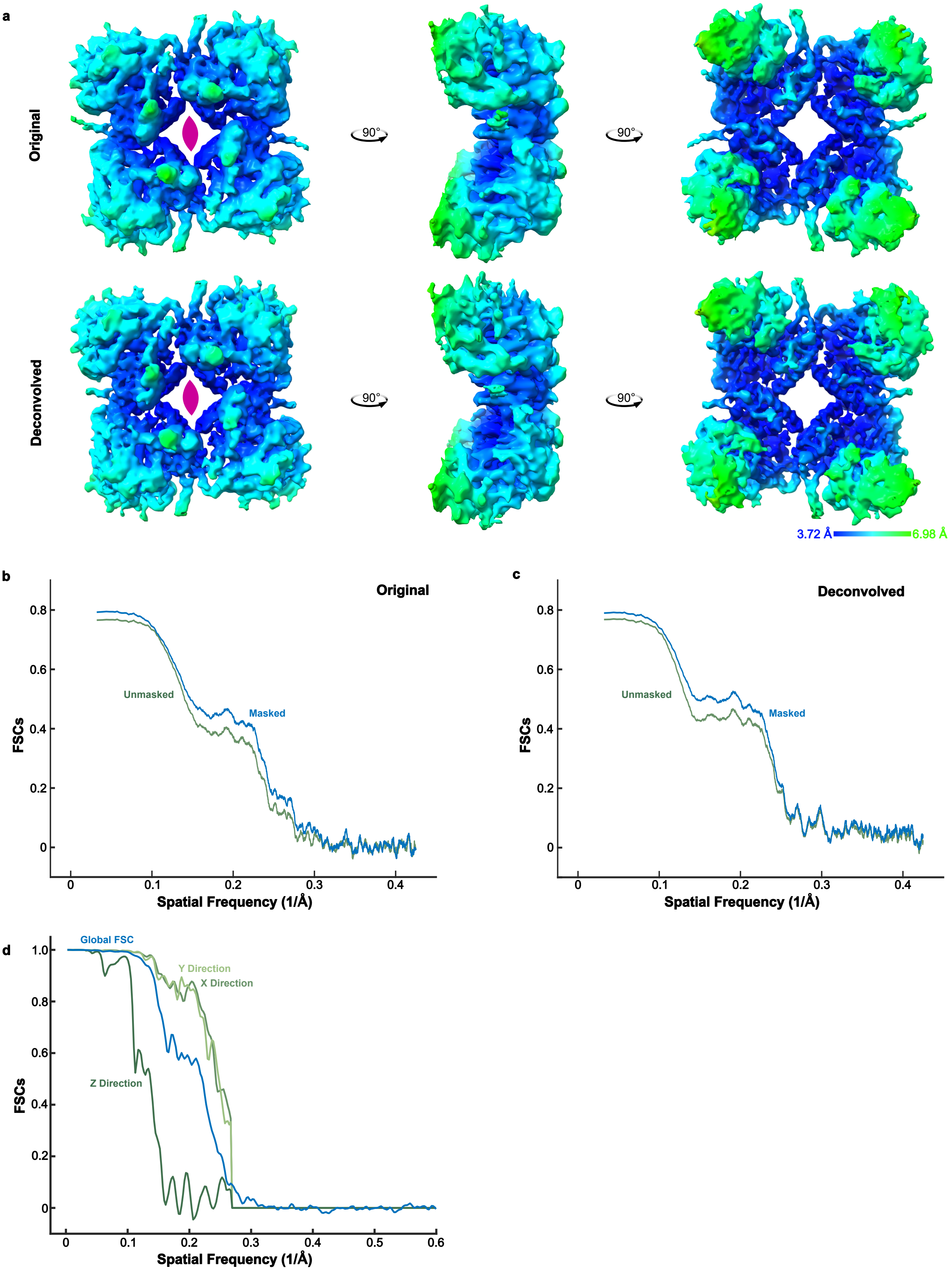


**Supplementary Fig. 6 | FSC curves corresponding to the original and deconvolved PhuN cryoEM maps. a,** Original and deconvolved maps colored by the local resolution of the original map. **b,c,** Map to model FSC for the original and deconvolved maps, respectively. **d.** 3DFSC calculated for the original map.

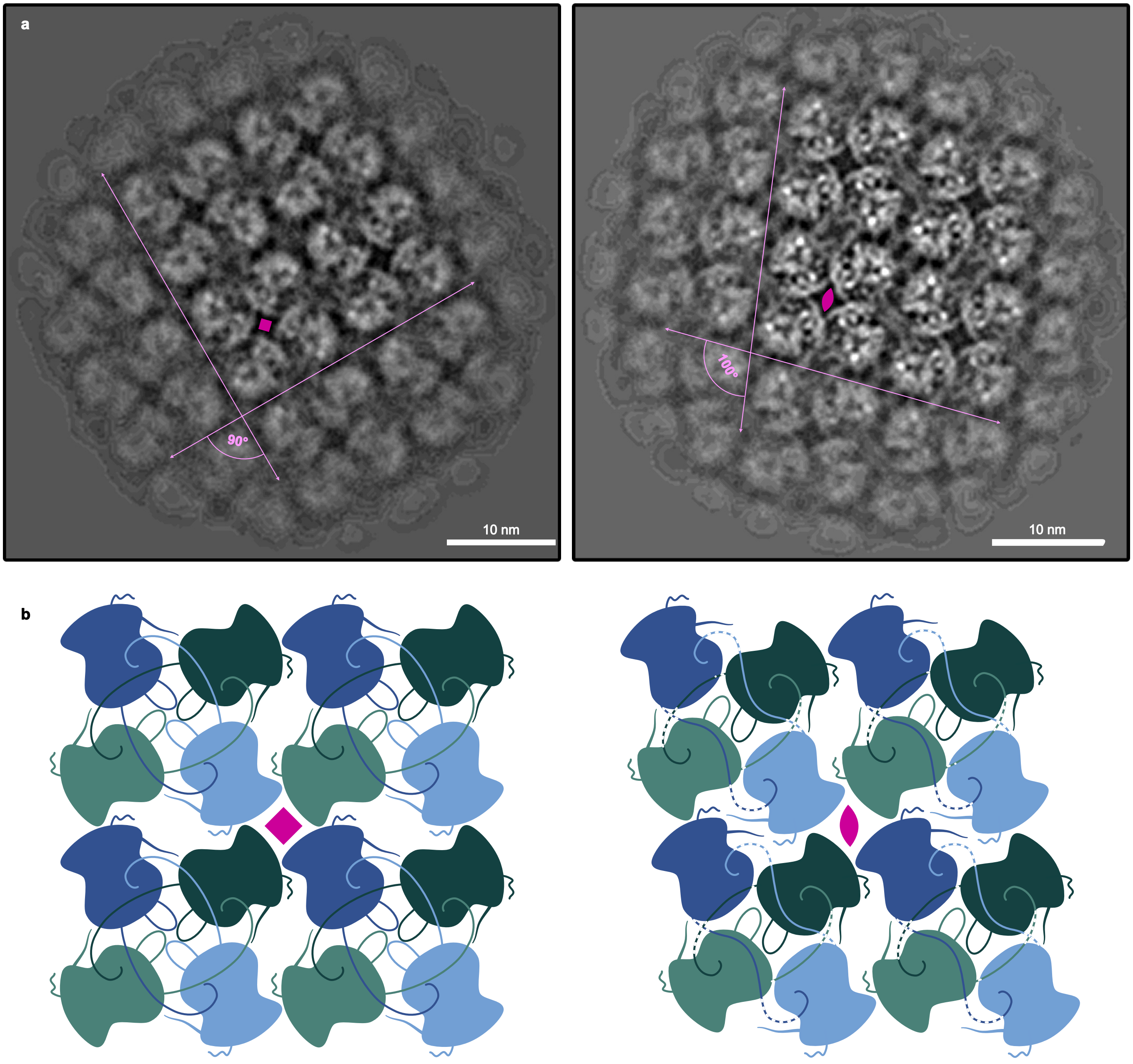


**Supplementary Fig. 7 | Comparison of p2 and p4 lattice symmetries. a,** A small population with p4 symmetry (left) is observed in 2D classification. The flexible loops at the Loop interface appear to all face the center while the remaining two interfaces are replaced by one that is most similar to the Open Hairpin interface in our p2 classes (right, full view of the class shown in Fig. 2a). **b,** Cartoon depiction of how the p4 and p2 lattices may compare.

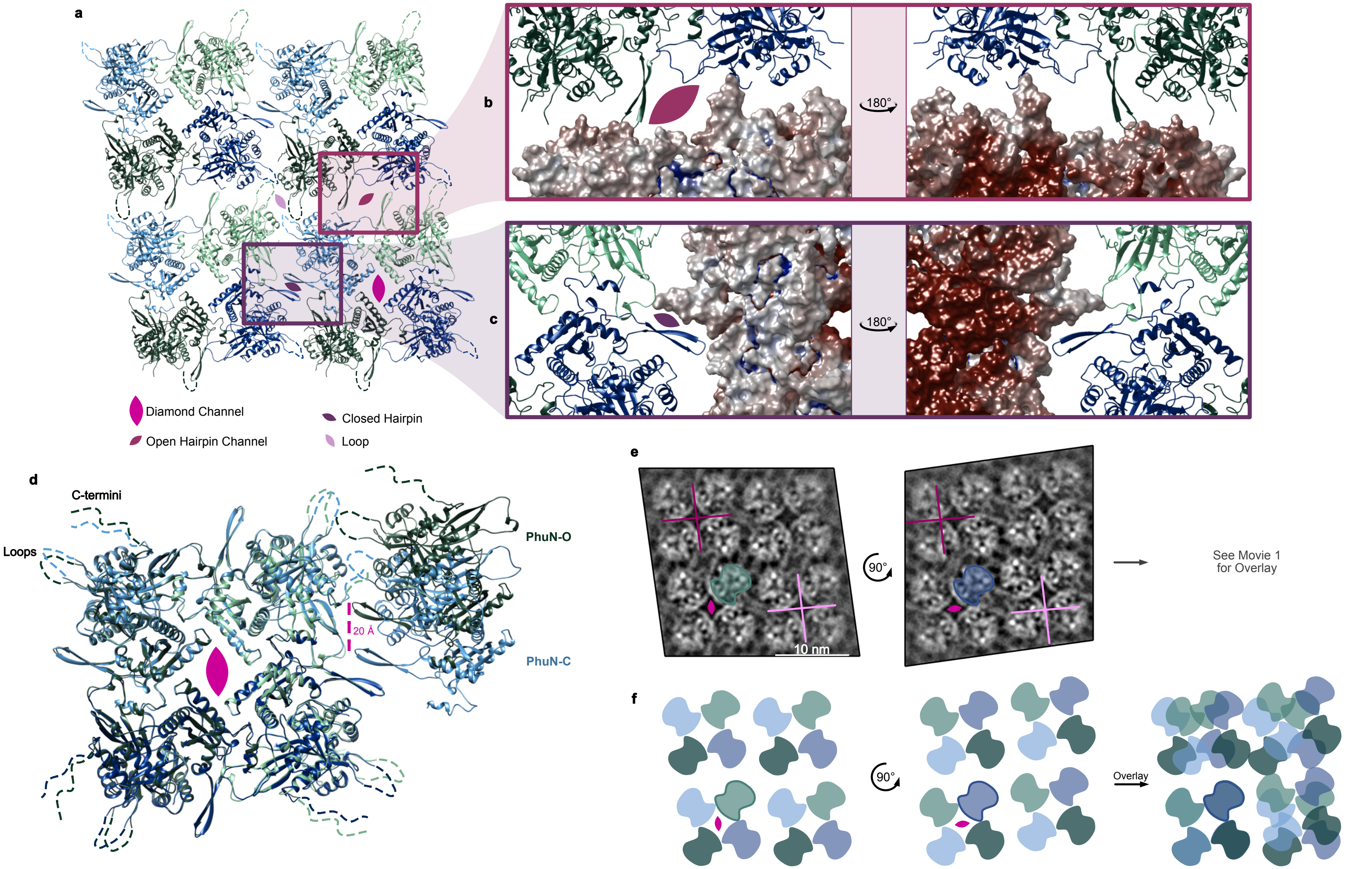


**Supplementary Fig. 8 | Closer look at the positioning of the β-Hairpin at the Open Channel and Closed Interface. a,** 16-mer model as in Fig. 2e. The two channels and two interfaces are labeled. **b,** The Open Channel is shown as viewed from the cytosol (left) and inside the phage nucleus (right). **c,** The Closed interface is similarly represented with the phage nucleus exterior on the left and interior on the right. The surfaces of two of the four subunits at each interface are shown and colored by electrostatic potential. **d,** Two tetramers aligned on the asymmetric subunits with a neighboring PhuN-O and PhuN-C subunit. The β-hairpin of PhuN-O is near the C-terminus of its neighbor while the PhuN-C β-hairpin is closer to a structured loop. **e,** 2D class, as shown in figure 2a, compared to a 90° rotation of the same 2D class after alignment on the highlighted asymmetric PhuN-O (green) and PhuN-C (blue) subunits. The centers of two Diamond Channels are shown with a light and dark pink cross (left) and are kept in the same position in the rotated image (right) to show the ~ 20 Å lattice shift. See Movie1 for a morph between the aligned subunits. **f,** Cartoon depiction of panel (e) with a direct overlay of the rotated lattice showing the new positioning of the shifted subunits. The alignment was done using the bolded PhuN-O (green) and PhuN-C (blue) as in panel (e).

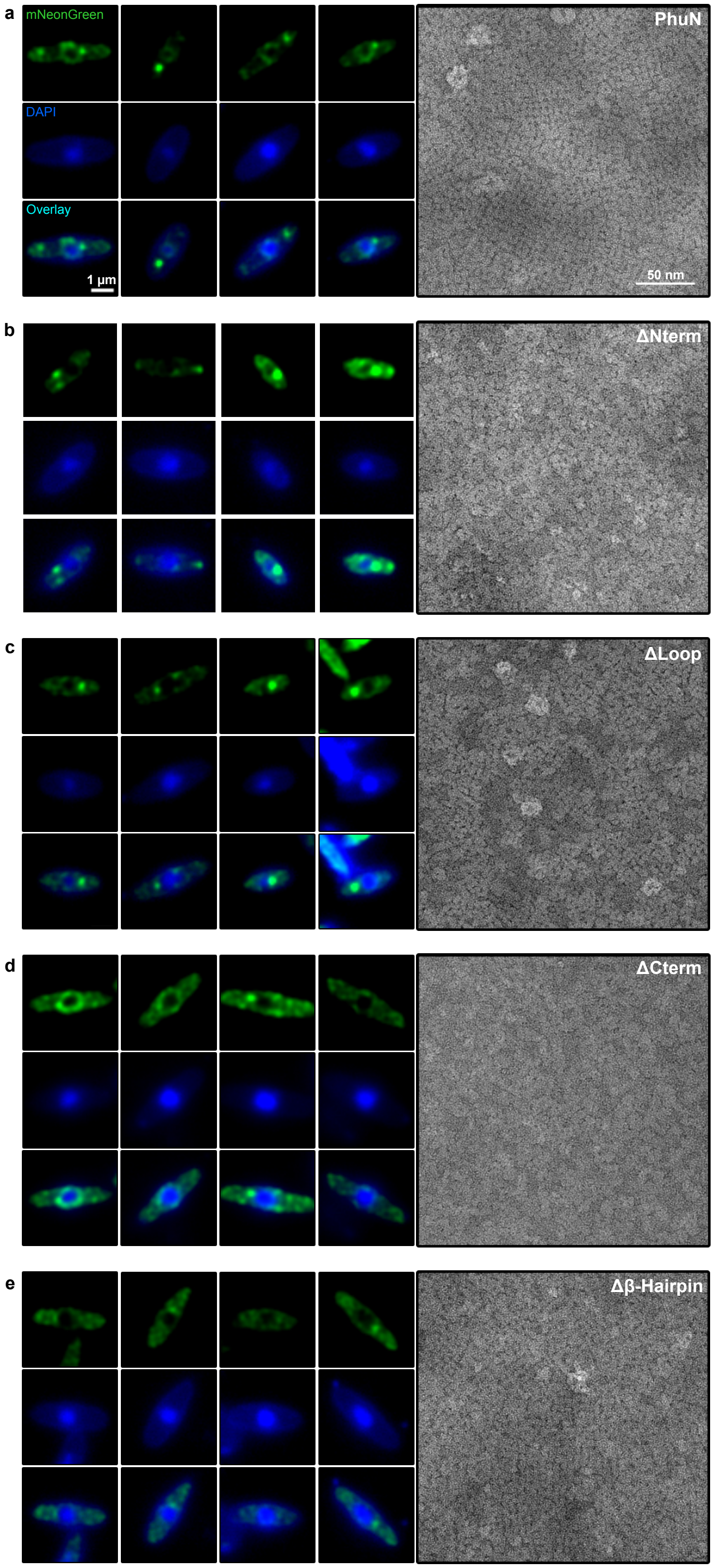


**Supplementary Fig. 9 | Additional images of ϕPA3 PhuN truncations showing defects in shell integration *in vivo* and self-assembly *in vitro*.** Live fluorescence microscopy and negative stain EM micrographs of **a,** full-length PhuN as well as the following PhuN deletions: **b,** N-terminal tail (residues 1-37), **c,** Loop (residues 272-291), **d,** C-terminal tail (residues 556-602), and **e,** β-Hairpin (residues 111-126). Fluorescence microscopy images of PhuN or PhuN deletions were collected at 40 minutes post infection, deconvolved, and are displayed in green while the the DAPI stained DNA is shown in blue. The 1 μm and 50 nm scale bars apply to all fluorescence microscopy and EM panels, respectively. (Independent sample preparation and imaging: fluorescence microscopy n = 3, negative stain EM n = 1).

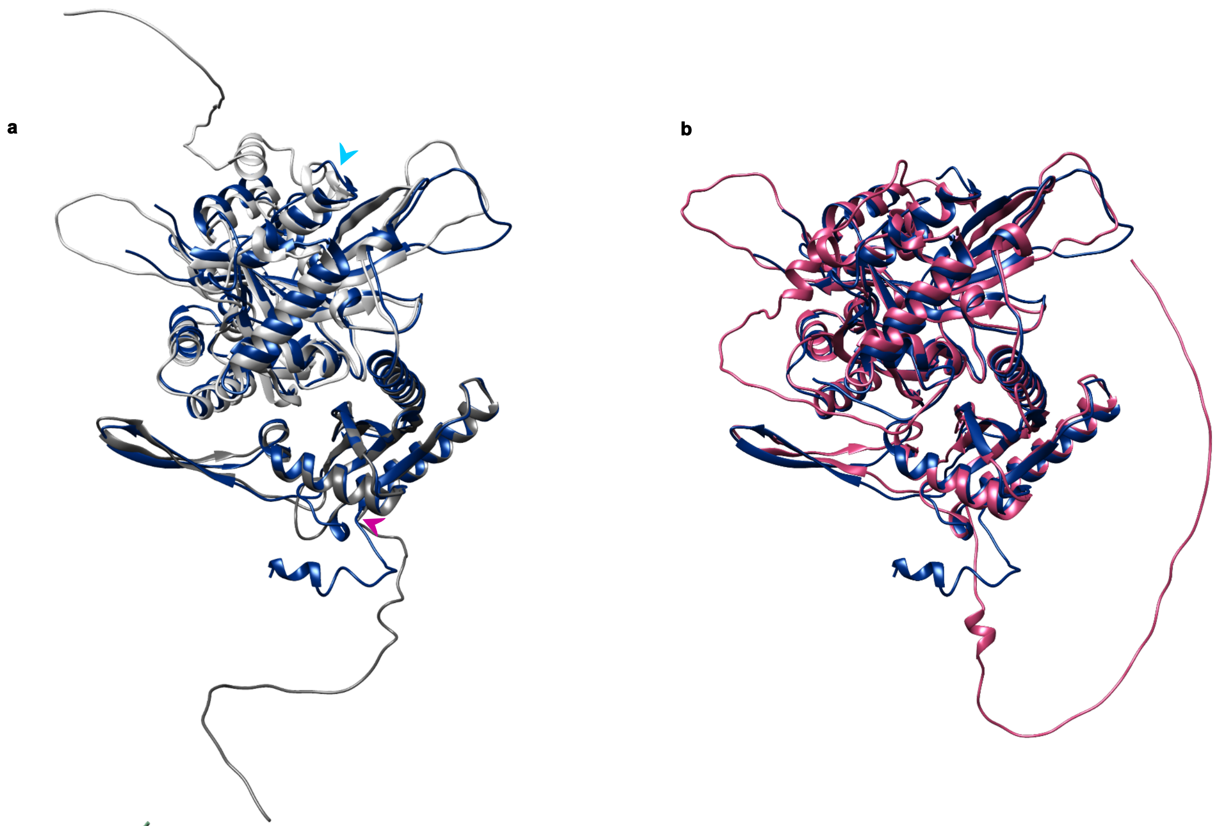


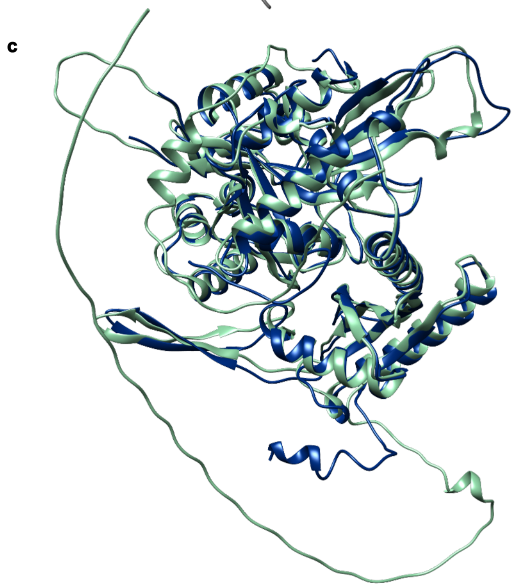


| d | ϕPA3 | ϕKZ | 201ϕ2-1 |
|---|---|---|---|
| N-terminal Residues | 36 | 53 | 64 |
| C-terminal Residues | 50 | 69 | 50 |

**Supplementary Fig. 10 | AlphaFold PhuN predictions compared to the final ϕPA3 PhuN model.** Final model of ϕPA3 gp53 (dark blue) overlaid with **a,** the starting ϕPA3 AlphaFold Prediction (grey), **b,** ϕKZ AlphaFold prediction (pink), and **c,** the 201ϕ2-1 AlphaFold prediction (green). In (b) and (c), the C-terminal tails are positioned across the back of the large, acetyltransferase-like domain. **d,** A summary of differing N- and C-terminal tail lengths as measured to the start of the helix lining the Diamond channel (pink arrow in panel a) for the N-term and from the end of the β-strand for the C-term (blue arrow in panel a).

| | **phiPA3 PhuN Tetramer, p2** (EMDB-29550) | **Deconvolved phiPA3 PhuN Tetramer, p2** (EMD-29310); **phiPA3 PhuN Tetramer, p2** (PDB 8FNE) | **Tracing p2 phiPA3 PhuN Tetramer Interfaces** (EMD-29451); **Representation of 16-mer phiPA3 PhuN Lattice, p2** (PDB 8FV5) |
|---|---|---|---|
| **Data collection and processing** | | | |
| Magnification | 105 kx | | |
| Voltage (kV) | 300 | | |
| Electron exposure (e–/Å²) | 66 or 67 | | |
| Defocus range (μm) | 0.8-2.5 | | |
| Pixel size (Å) | 0.834-0.835 | | |
| Symmetry imposed | C2 | | |
| Initial particle images (no.) | 600,734 | | |
| Final particle images (no.) | 29,303 | | 161,270 |
| Map resolution (Å)<br>FSC threshold | 3.9<br>0.143 | - | 7.0<br>0.143 |
| Map resolution range (Å) | 3.72-6.98 | | 4.52-7.95 |
| **Refinement** | | | |
| Initial model used | AlphaFold Prediction | | - |
| Model resolution (Å)<br>FSC threshold | 6.8<br>0.5 | 6.9<br>0.5 | - |
| Map sharpening *B* factor (Å²) | - | 80 | - |
| Model composition<br>Non-hydrogen atoms<br>Protein residues | 16,864<br>2,148 | | - |
| R.m.s. deviations<br>Bond lengths (Å)<br>Bond angles (°) | 0.018<br>1.748 | | - |
| Validation<br>MolProbity score<br>Clashscore<br>Poor rotamers (%) | 0.60<br>0.27<br>0.00 | | - |
| Ramachandran plot<br>Favored (%)<br>Allowed (%)<br>Disallowed (%) | 98.96<br>1.04<br>0.00 | | - |

**Supplementary Table 1 | Cryo-EM data collection, refinement, and validation statistics**

| Collected Datasets by Grid Type, Tilt, and Frame Rate | | | | | | |
|---|---|---|---|---|---|---|
| **Grid Type** | **Quantifoil** | **GO/$NH_2$** | **GO/$NH_2$** | **GO/$NH_2$** | **UltrAuFoil** | **UltrAuFoil GO/$NH_2$** |
| **Tilt Angle** | 0°, 40°, 50° | 0°, 60° | 0°, 15° | 0°, 30°, 45°, 60° | 0°, 60° | 35° |
| **Frames** | 120 | 118 | 246 | 444 | 444 | 444 |
| **Magnification** | 105,000 | | | | | |
| **Voltage (kV)** | 300 | | | | | |
| **Electron Dose (e-/Å$^2$)** | 66 | 67 | 67 | 67 | 67 | 67 |
| **Defocus Range (μm)** | 1.5-2.5 | 0.8-1.6 | 0.8-1.6 | 0.8-1.6 | 0.8-1.6 | 0.8-1.6 |
| **Pixel Size (Å)** | 0.834 | 0.835 | 0.835 | 0.835 | 0.835 | 0.835 |

**Supplementary Table 2 | Cryo-EM data collection parameters**

| NTD | | | | | | |
| --- | --- | --- | --- | --- | --- | --- |
| PDB ID-Chain | Z | rmsd | lali | nres | %ID | PDB Description |
| 3s1s-A | 5.5 | 3.1 | 96 | 871 | 7 | RESTRICTION ENDONUCLEASE BPUSI |
| 3nat-A | 5.2 | 3.4 | 98 | 156 | 14 | UNCHARACTERIZED PROTEIN |
| 2w8m-B | 4.7 | 4.5 | 101 | 168 | 11 | ORF D212 |
| 6em5-b | 4.6 | 3.7 | 98 | 421 | 15 | 5.8S RIBOSOMAL RNA |
| 3nbm-A | 4.5 | 3.1 | 80 | 104 | 6 | PTS SYSTEM, LACTOSE-SPECIFIC IIBC COMPONENTS |
| 1yra-B | 4.5 | 3.3 | 101 | 261 | 9 | ATP(GTP)BINDING PROTEIN |
| 6vda-A | 4.5 | 3.1 | 67 | 86 | 10 | ZT_DIMER DOMAIN-CONTAINING PROTEIN |
| 2eo0-A | 4.5 | 3.4 | 89 | 130 | 7 | HYPOTHETICAL PROTEIN ST1444 |
| 2ayx-A | 4.4 | 3.1 | 84 | 254 | 10 | SENSOR KINASE PROTEIN RCSC |
| 1d02-B | 4.4 | 3.9 | 96 | 200 | 11 | DNA (5'-D(*GP*CP*CP*AP*AP*TP*TP*GP*GP*C)-3') |
| 6xpd-A | 4.3 | 3.7 | 76 | 303 | 3 | ZINC TRANSPORTER 8 |
| 6vd8-B | 4.3 | 3.7 | 63 | 78 | 6 | PROBABLE CATION EFFLUX SYSTEM PROTEIN |
| 2vyc-A | 4.3 | 3.8 | 96 | 755 | 7 | BIODEGRADATIVE ARGININE DECARBOXYLASE |
| 1s28-A | 4.2 | 3.2 | 79 | 130 | 6 | ORF1 |
| 5fr7-B | 4.1 | 2.9 | 77 | 146 | 12 | AMYR |
| 4ccs-A | 4.1 | 3.5 | 93 | 226 | 6 | CBIX |
| 7p82-C | 4.1 | 5.0 | 89 | 408 | 3 | S-ADENOSYLMETHIONINE SYNTHASE |
| 5u4u-A | 4.0 | 3.5 | 91 | 157 | 5 | MGC81300 PROTEIN |
| 6jcx-F | 4.0 | 3.0 | 66 | 186 | 9 | DNA-DIRECTED RNA POLYMERASE SUBUNIT ALPHA |
| 1hrk-A | 4.0 | 4.4 | 100 | 359 | 7 | FERROCHELATASE |

| CTD | | | | | | |
| --- | --- | --- | --- | --- | --- | --- |
| PDB ID-Chain | Dali Z-score | rmsd | lali | nres | %id | PDB Description |
| 7byy-A | 5.7 | 3.9 | 134 | 180 | 9 | ACETYLTRANSFERASE |
| 1y9k-B | 5.2 | 3.6 | 118 | 154 | 8 | IAA ACETYLTRANSFERASE |
| 6c30-A | 5.1 | 3.4 | 129 | 213 | 8 | GNAT FAMILY ACETYLTRANSFERASE |
| 3f5b-A | 5.1 | 3.0 | 120 | 172 | 6 | AMINOGLYCOSIDE N(6')ACETYLTRANSFERASE |
| 1kzf-A | 5.0 | 4.3 | 127 | 198 | 9 | ACYL-HOMOSERINELACTONE SYNTHASE ESAI |
| 1xeb-A | 5.0 | 3.9 | 112 | 149 | 8 | HYPOTHETICAL PROTEIN PA0115 |
| 2vzz-A | 4.6 | 3.5 | 126 | 210 | 6 | RV0802C |
| 7kd7-A | 4.6 | 3.9 | 124 | 204 | 9 | N-ALPHA-ACETYLTRANSFERASE 40 |
| 3owc-A | 4.5 | 3.8 | 120 | 172 | 10 | PROBABLE ACETYLTRANSFERASE |
| 7cs1-A | 4.5 | 4.5 | 121 | 191 | 10 | AMINOGLYCOSIDE 2'-N-ACETYLTRANSFERASE |
| 5hgz-A | 4.4 | 4.2 | 124 | 211 | 6 | N-ALPHA-ACETYLTRANSFERASE 60 |
| 6edw-B | 4.3 | 3.8 | 122 | 746 | 11 | ISOCITRATE LYASE 2 |
| 5ktc-A | 4.3 | 3.5 | 119 | 187 | 10 | FDHC |
| 3efa-A | 4.2 | 3.5 | 108 | 146 | 3 | PUTATIVE ACETYLTRANSFERASE |
| 5o7o-A | 4.1 | 3.0 | 108 | 146 | 6 | DESFERRIOXAMINE SIDEROPHORE BIOSYNTHESIS PROTEIN |
| 2wpw-A | 4.0 | 4.3 | 129 | 328 | 5 | ORF14 |
| 5gnc-A | 3.9 | 12.1 | 72 | 597 | 6 | AVH146 |
| 3fnc-B | 3.8 | 3.5 | 111 | 161 | 6 | PUTATIVE ACETYLTRANSFERASE |
| 4rs2-B | 3.8 | 3.9 | 112 | 183 | 6 | PREDICTED ACYLTRANSFERASE WITH ACYL-COA N-ACYLTRA |
| 3t9y-A | 3.8 | 3.1 | 95 | 135 | 9 | ACETYLTRANSFERASE, GNAT FAMILY |

**Supplementary Table 3 | Dali Server searches of the PhuN NTD and CTD.**
20 highest scoring Dali Server outputs with the NTD and CTD as the input.
