# Supplementary material for: The ϕPA3 phage nucleus is enclosed by a self-assembling 2D crystalline lattice
Source: Nat Commun. 2023 Feb 18;14:927. doi: 10.1038/s41467-023-36526-9 (PMC9938867; doi:10.1038/s41467-023-36526-9)
Supplement: Supplementary file 3 — Description of Additional Supplementary Files [file 41467_2023_36526_MOESM3_ESM.pdf]

File name: Supplementary Movie 1

Description: Movie showing a morph between the same 2D class rotated by ~ 90° after aligning on the asymmetric PhuN-O and PhuN-C subunits. The ~ 20 Å lattice/β-hairpin shift is visible.
