# Supplementary material for: The ϕPA3 phage nucleus is enclosed by a self-assembling 2D crystalline lattice
Source: Nat Commun. 2023 Feb 18;14:927. doi: 10.1038/s41467-023-36526-9 (PMC9938867; doi:10.1038/s41467-023-36526-9)
Supplement: Supplementary file 5 — Reporting Summary [file 41467_2023_36526_MOESM5_ESM.pdf]

Corresponding author(s): David A. Agard

Last updated by author(s): January 16, 2022

Reporting Summary

Nature Portfolio wishes to improve the reproducibility of the work that we publish. This form provides structure for consistency and transparency in reporting. For further information on Nature Portfolio policies, see our Editorial Policies and the Editorial Policy Checklist.

Statistics

For all statistical analyses, confirm that the following items are present in the figure legend, table legend, main text, or Methods section.

n/a	Confirmed	
☐	☒	The exact sample size (n) for each experimental group/condition, given as a discrete number and unit of measurement
☒	☐	A statement on whether measurements were taken from distinct samples or whether the same sample was measured repeatedly
☒	☐	The statistical test(s) used AND whether they are one- or two-sided<br>*Only common tests should be described solely by name; describe more complex techniques in the Methods section.*
☒	☐	A description of all covariates tested
☒	☐	A description of any assumptions or corrections, such as tests of normality and adjustment for multiple comparisons
☒	☐	A full description of the statistical parameters including central tendency (e.g. means) or other basic estimates (e.g. regression coefficient) AND variation (e.g. standard deviation) or associated estimates of uncertainty (e.g. confidence intervals)
☒	☐	For null hypothesis testing, the test statistic (e.g. F, t, r) with confidence intervals, effect sizes, degrees of freedom and P value noted<br>*Give P values as exact values whenever suitable.*
☒	☐	For Bayesian analysis, information on the choice of priors and Markov chain Monte Carlo settings
☒	☐	For hierarchical and complex designs, identification of the appropriate level for tests and full reporting of outcomes
☒	☐	Estimates of effect sizes (e.g. Cohen's d, Pearson's r), indicating how they were calculated

Our web collection on statistics for biologists contains articles on many of the points above.

Software and code

Policy information about availability of computer code

Data collection	cryoEM data for structure determination was collected using SerialEM (v.3.6-v.3.8). Live fluorescence microscopy was done using Nikon Elements AR software (version 5.02.00).
Data analysis	cryoSparc(v2.15.0, v3.3.1); Relion (V.3.0.8); UCSF MotionCor2; cisTEM(1.0.0-beta); Chimera(1.13.1); ChimeraX-1.2.1; ChimeraX-Isolde 1.2.2; PHENIX (v1.20.1-4487-000); 3dmod Version 4.11.20; RoseTTAFold (pre-release); RoseTTA v.3.11; ER-Decon II; PRIISM; Huygens Essential 20.10 (Scientific Volume Imaging, the Netherlands, http://svi.nl), Nikon Elements AR software (version 5.02.00); Fiji version 2.1.0/1.53c, AlphaFold2 w/Jackhmmer; MatLab (v.9.11.0 and v.9.12.0); PRIISM and ER-Decon II are both available on request from agard@msg.ucsf.edu as the terms of the license established prior to this study with the University of California and HHMI do not allow uploading the code in a public repository.

For manuscripts utilizing custom algorithms or software that are central to the research but not yet described in published literature, software must be made available to editors and reviewers. We strongly encourage code deposition in a community repository (e.g. GitHub). See the Nature Portfolio guidelines for submitting code & software for further information.

Data

Policy information about availability of data

All manuscripts must include a data availability statement. This statement should provide the following information, where applicable:

- Accession codes, unique identifiers, or web links for publicly available datasets
- A description of any restrictions on data availability
- For clinical datasets or third party data, please ensure that the statement adheres to our policy

The cryoEM maps generated in this study have been deposited to the Electron Microscopy Data Bank (EMDB) under accession codes EMD-29550 [https://www.ebi.ac.uk/emdb/entry/EMD-29550] (phiPA3 PhuN Tetramer, p2), EMD-29310 [https://www.ebi.ac.uk/emdb/entry/EMD-29310] (Deconvolved phiPA3 PhuN Tetramer, p2), and EMD-29451 [https://www.ebi.ac.uk/emdb/entry/EMD-29451] (Tracing p2 phiPA3 PhuN Tetramer Interfaces). The atomic coordinates have been deposited to the PDB under accession codes 8FNE [http://doi.org/10.2210/pdb8fne/pdb] (phiPA3 PhuN Tetramer, p2) and 8FV5 [http://doi.org/10.2210/pdb8fv5/pdb] (Representation of 16-mer phiPA3 PhuN Lattice, p2). Publicly available entries used in this study are PDB 7SQR and EMDB EMD-25221. All other data, bacterial strains, and plasmids are available from the corresponding author on a reasonable request.

Human research participants

Policy information about studies involving human research participants and Sex and Gender in Research.

Reporting on sex and gender	N/A
Population characteristics	N/A
Recruitment	N/A
Ethics oversight	N/A

Note that full information on the approval of the study protocol must also be provided in the manuscript.

Field-specific reporting

Please select the one below that is the best fit for your research. If you are not sure, read the appropriate sections before making your selection.

☒ Life sciences ☐ Behavioural & social sciences ☐ Ecological, evolutionary & environmental sciences

For a reference copy of the document with all sections, see nature.com/documents/nr-reporting-summary-flat.pdf

Life sciences study design

All studies must disclose on these points even when the disclosure is negative.

Sample size	No sample size calculation was performed. For cryoEM reconstructions, data was collected in accordance with microscope availability and need to sample side-views (0-60 degree tilts) as judged by changes in resolution/z-stretch of the map. Sample sizes for other experiments were determined by instrument and resource availability as well as observing the same phenotypes consistently. Specifically, live fluorescence microscopy imaging sample sizes were determined by observing a consistent range of phenotypes repeatedly in at least n = 200 cells per imaged construct and are represented in Supplementary Figure 10.
Data exclusions	Data was excluded as with accepted single particle cryoEM processing pipelines. 2D averages were used to keep or exclude data, followed by 3D classification to further remove low resolution or poorly aligned particles.
Replication	2D lattices were obtained at least three times in negative stain with protein alone over two years using protein from independent purifications. 2D lattices assembled using a lipid monolayer were obtained at least four separate times in cryoEM over 10 months and again two years later. MBP-PhuN immunofluorescence sample preparation and imaging was done once. Phage nuclear fragment isolations were done once each with and without the MBP tag for cryoEM. The isolations were repeated twice for negative stain EM with the MBP tag. All attempts at replication have been successful, however, isolating what we believe to be intact compartments is rare likely as a result of mechanical stress during the purification process. Deletions were imaged on three separate occasions within months of each other, viewing at least n = 200 cells for each deletion. All attempts at replicating the live imaging have been successful.
Randomization	Experimental groups were not allocated and thus no randomization was performed.
Blinding	Investigators were not blinded for any part of the experiments as this is not a common or advantageous practice for cryoEM. As cryoEM is a method of direct sample visualization, it's important to closely monitor the data processing and be well informed while making decisions in the processing workflow. While no blinding was done, the initial particle selection, 2D classification, and structure determination were completed via template-free and ab-initio workflows.

Reporting for specific materials, systems and methods

We require information from authors about some types of materials, experimental systems and methods used in many studies. Here, indicate whether each material, system or method listed is relevant to your study. If you are not sure if a list item applies to your research, read the appropriate section before selecting a response.

Materials & experimental systems

n/a	Involved in the study
☐	☒ Antibodies
☒	☐ Eukaryotic cell lines
☒	☐ Palaeontology and archaeology
☒	☐ Animals and other organisms
☒	☐ Clinical data
☒	☐ Dual use research of concern

Methods

n/a	Involved in the study
☒	☐ ChIP-seq
☒	☐ Flow cytometry
☒	☐ MRI-based neuroimaging

Antibodies

Antibodies used	Maltose Binding Protein Epitope Tag Antibody (Rockland Inc. Rabbit Polyclonal, Product # 200-401-385) used at 2 ug/mL; ThermoFisher Goat Anti-rabbit IgG (H+L) Highly Cross-Adsorbed Secondary Antibody, Alexa Fluor555 (Catalog #A-21429) used at 4 ug/mL
Validation	Purity/Specificity Statement directly quoted from Rockland: "This IgG purified antibody is directed against MBP and is useful in determining its presence in various assays. This polyclonal anti-MBP tag antibody detects over-expressed proteins containing the MBP epitope tag. To date this antibody has reacted with all MBP tagged proteins so far tested. In western blotting of bacterial extracts the antibody does not cross-react with endogenous proteins." The Secondary Antibody has been prepared by Invitrogen using affinity purification to remove cross-reactive species and has been validated with immunofluorescence analysis by Invitrogen; it has also been used extensively in publications (559 references cited by ThermoFisher to date).
